# Living Conditions and Social Outcomes in Adults With Cerebral Palsy

**DOI:** 10.3389/fneur.2021.749389

**Published:** 2021-10-21

**Authors:** Katina Pettersson, Elisabet Rodby-Bousquet

**Affiliations:** ^1^Centre for Clinical Research, Uppsala University/Region Västmanland, Västerås, Sweden; ^2^Department of Clinical Sciences, Lund University, Lund, Sweden

**Keywords:** cerebral palsy, adults (MeSH), domestic partners, employment, housing, occupation, personal assistance, social security

## Abstract

**Objectives:** To analyse the living conditions and social outcomes (housing, engagement in employment or higher education, access to personal assistance and having a partner) in adults with cerebral palsy (CP) relative to their age, sex, communication ability, and motor skills.

**Methods:** Cross-sectional registry-based study of 1,888 adults (1,030 males/858 females) with CP in the Swedish CP follow-up programme, median age 25 years (range 16–78 y). Type of housing, occupation, access to personal assistance and having a partner were analysed relative to their age, sex, and the classification systems for Gross Motor Function (GMFCS) and Communication Function (CFCS). Binary logistic regression models were used to calculate odds ratios (OR) for independent living, competitive employment, and having a partner.

**Results:** Most of the 25- to 29-year olds (55.6%) lived independently, increasing to 72.4% in 40- to 49-year olds, while the majority (91.3%) of those under 20 years lived with their parents. Independent living was almost equal in adults at GMFCS levels I (40.2%) and V (38.6%). This parity was explained by access to personal assistance, which increased with higher GMFCS and CFCS levels. Personal assistance of >160 hours/week was associated with a high probability of independent living (OR 57). In the age span 20–64 years, 17.5% had competitive employment and 45.2% attended activity centres for people with intellectual disabilities. In the younger age group up to 24 years old, 36.9% went to mainstream/higher education and 20.5% went to special schools. In total, 13.4% had a partner and 7.8% lived together. Slightly more women than men had a partner, and most individuals were classified at CFCS level I.

**Conclusion:** Only one in eight adults with CP has a partner, and one in six has competitive employment. Access to personal assistance is the single most important factor for independent living. It is vital to support adults with CP throughout their lifespan to achieve the best possible outcomes in all aspects of life.

## Introduction

Today, most adults with cerebral palsy (CP) have a full life expectancy (1). The prevalence of CP is estimated to be 17 million individuals worldwide (2). Those with the most severe impairments have poor survival, but the surviving adults have significantly fewer impairments, particularly regarding severe motor impairment, intellectual disability and epilepsy (3). Even so, our knowledge of their living conditions and social outcomes is sparse.

Qualitative studies of young adults with CP show that living arrangements, occupation, personal care and interpersonal relationships are among the most important issues to address (4–6). According to the World Health Organisation, social well-being is an important aspect of health (7). Individuals with neurodevelopmental conditions such as CP are less likely to pursue post-secondary education. They also experience lower employment rates and participate in fewer leisure and social activities. In daily life, they rely more on their families for living arrangements, and adults with CP experience poorer health than their peers without disabilities. There is still a great need to understand better the development of individuals with CP through their lifetime (8). Population-based studies may increase our understanding of the living conditions of adults with CP, leading to knowledge that facilitates the distribution of resources (3).

To improve the continuity of care throughout life and bridge the gap between paediatric and adult care, a follow-up programme for adults with CP (CPUP) was initiated as a pilot project in Sweden in 2009. The programme has expanded and since 2019, all 21 health-care regions in Sweden offer ongoing follow-up within this programme. The transition from child- to adult services within the programme is flexible from 16 up to 19 years. The programme originally started in 1994 as hip surveillance of children (9) and over the years has developed into a National multiprofessional health-care programme and quality registry. Now, the programme involves orthopaedic surgeons and hand surgeons, neuropaediatricians, physical and occupational therapists, speech and language pathologists, psychologists and certified prosthetists and orthotists. Since 1994, Swedish law has decreed that certain physically disabled people over the age of 20 years have the right to services by specialists and to rehabilitation in special units. Before then, only individuals over the age of 20 years with learning disabilities were guaranteed services and rehabilitation (10).

An early study of the adult population with CP within the follow-up programme showed that most individuals lived at home with their parents. They either studied or had their occupation at activity centres; 34 out of 70 young adults had personal assistance. The study included 102 individuals in their early twenties, explaining the high number of individuals living at home and in higher education (11). A Danish study showed that 55% of Danish adults with CP (aged 29–35 years) were unemployed, did not cohabit with a partner and did not have children, compared with only 4% of the control population (12). Andersson and Mattsson (10) reported results from a Swedish population of 121 adults with CP (20–58 years) with most adults living independently and in single households, with or without home services and that 24% worked full time. A study from the Netherlands reported employment rates of 49% in 74 young adults (20–years) with spastic CP without intellectual disabilities (13), which is slightly higher than the 18% recently reported in a Swedish study of 61 young adults with CP (20–22 years old) (6).

The aim of this study was to analyse the living conditions and social outcomes (housing, engagement in employment or higher education, access to personal assistance, and having a partner) in adults with CP relative to their age, sex, communication ability and motor function.

## Materials and Methods

### Participants

This cross-sectional registry-based study included all adults followed within the Swedish CP follow-up program from 2012 until 2019. The age ranged from 16 to 78 years and a majority of those included in the program were enrolled as adults and have not previously been followed as children. All participation was voluntary, and all participants gave their consent, even if they previously had participated in the follow-up as children. The male/female ratio corresponded to the CP prevalence in children with slightly more males than females.

The adults were examined regularly by their local adult specialist team, (usually a physical therapist, an occupational therapist and a speech and language pathologist), according to a schedule based on their level of the Gross Motor Function Classification System (GMFCS). Adults classified at levels III to V are offered examinations every year and those at levels II and I are examined every second or third year. The examinations are accompanied by several patient-reported outcome measures and information about living conditions and social outcomes.

### Classifications and Measurements

The CP definition used was that of Rosenbaum et al. (14). The inclusion and exclusion criteria for CP was defined according to the Surveillance of Cerebral Palsy in Europe as a brain injury before the age of 2 years, with subtypes divided into unilateral spastic CP, bilateral spastic CP, ataxic CP, dyskinetic CP or mixed type/unclassifiable CP (15). Functional levels were classified by the local specialist team according to the expanded and revised version of the GMFCS, describing gross motor performance (16), and the Communication Function Classification System (CFCS), which describes the effectiveness of communication as a sender and receiver of information including all types of communication such as facial expressions and alternative communication (17).

### Sex and Age

Sex was based on the legal gender, male or female. Age at examination was calculated based on the date of birth and date of examination. Age was grouped into seven categories: 16–19, 20–24, 25–29, 30–39, 40–49, 50–64, and 65–78 years. The rationale for this grouping was the skewed distribution due to an excess of younger people, where people in the transition years are more likely to attend school and live with their parents. The formative years, where many are moving into higher education, starting to work and moving away from home were divided into several age categories. There is no fixed retirement age in Sweden, but the age for the guaranteed pension is 65 years, therefore adults 65 years and older were grouped together. Guaranteed pension includes people with disabilities and those who never worked.

### Having a Partner

Having a partner was categorised as (1) Single, (2) Partner who lives elsewhere, or (3) Domestic partner (reside together with partner or spouse).

### Type of Housing

The participants' type of housing was categorised as (1) independent living (own housing, with or without assistance); (2) living with parents; (3) assisted-living facilities (e.g., group homes and service housing provided by the municipality); or (4) other living arrangements.

### Personal Assistance

Data on personal assistance were collected and divided into the following categories, depending on the hours of assistance per week (h/w): (1) <60, (2) 60–160, (3) >160 h/w, or (4) no assistance. The Swedish Personal Assistance act is demand-driven and entitles personal assistance (part-time or full time) to individuals with certain disabilities who need help more than 20 h/w for activities of daily living. The personal assistants are carers paid for by the state and the costs are fully covered. The need for assistance is expressed as h/w, and the needs are assessed by tax-funded Social Security. Eligibility is independent of income, property or housing of the person or their family. The individual is free to buy services from any provider or employ their own assistants (18).

### Occupation

Occupation status was classified as (1) mainstream education; (2) special school (schools for individuals with intellectual disabilities, the intelligence quotient (IQ) for such schooling is an IQ of <70); (3) competitive employment; (4) supported employment (for example, ventures stipulated by the government to offer an occupation to individuals outside the labour market); (5) activity centre (according to the Swedish law on special support and service for certain disabled people, daily activity can be provided to adults, 18–65 years old, with a developmental disorder, autism or autism-like condition or significant and permanent intellectual disability [IQ <70]. Activity centres are intended to give a meaningful work life and contribute to the development of the adult during weekdays, at so-called “day centres”. The activity offered is based on the individual's functional ability and interests and organised by the municipality); or (6) No occupation. The different categories of occupation were divided into full time (>30 h/w), or part-time ( ≤ 30 h/w).

### Ethics

This study was carried out in accordance with the Code of Ethics of the World Medical Association (Declaration of Helsinki) for experiments involving humans (19). The study was approved by the Medical Research Ethics Committee at Lund (no. 2009/341).

### Statistical Analyses

Frequencies and percentages were used to describe the data. The chi-squared test was used to detect any differences between categorical data. Binary logistic regression models were used to predict independent living, competitive employment and having a partner based on other variables such as age, sex, and functional levels. Age was used as a continuous variable. The results were presented as adjusted odds ratios (ORs) with 95% confidence intervals (CI). All values were adjusted for all other variables in the models. The significance level was set to 0.05. IBM SPSS Statistics for Windows, Version 26.0. Armonk, NY: IBM Corp was used for all analyses.

## Results

### Participants

Data from 1,888 adults with CP born between 1941 and 2003 were reported into the database, made up of 1,030 males (54.6%) and 858 females (45.4%), at a median age of 25 years (range 16–78 y) (Table 1). Four out of five individuals were younger than 40 years of age. Most adults were either classified at GMFCS level I (22.9%) or GMFCS level V (23.0%), with the fewest individuals at GMFCS level III (14.8%). Many of the adults had better communication than gross motor skills, and the majority were classified at CFCS level I (43.4%) or evenly distributed among CFCS levels II to V ranging from 11.8 to 14.3%. Spastic CP was the most frequent neurological subtype, with 983 (52.1%) having spastic bilateral CP and 444 (23.5%) spastic unilateral CP. Only 76 adults (4%) had ataxic CP and 233 (12.3%) had dyskinetic CP (Table 1).

**Table 1:** Characteristics of the 1,888 adults with CP.

		**Participants**	
		***N***	**%**
Total		1,888	100
**Sex**			
	Male	1,030	54.6
	Female	858	45.4
**Age groups**			
	16–19 y	355	18.8
	20–24 y	556	29.4
	25–29 y	344	18.2
	30–39 y	328	17.4
	40–49 y	165	8.7
	50–64 y	127	6.7
	65–78 y	13	0.7
**GMFCS**			
	I	432	22.9
	II	401	21.2
	III	279	14.8
	IV	341	18.1
	V	435	23.0
**CFCS**			
	I	819	43.4
	II	226	12.0
	III	225	11.9
	IV	222	11.8
	V	270	14.3
	Missing	126	6.7
**CP Subtype**			
	Spastic unilateral	444	23.5
	Spastic bilateral	983	52.1
	Ataxic	76	4.0
	Dyskinetic	233	12.3
	Mixed type/unclassifiable	121	6.4
	Missing	31	1.6

*CFCS, Communication Function Classification System; GMFCS, Gross Motor Function Classification System; Y, years*.

### Having a Partner

Most adults with CP were single (86.6%). Of the 13.4% who had a partner, 7.8% had a domestic partner and 5.6% had a partner who lived elsewhere (Table 2). More females (17.5%) than males (10%) had a partner, both a domestic partner (10.6 vs. 5.5%) and a partner living elsewhere (6.9 vs. 4.5%). Having a partner ranged from 2.1% of the youngest age group to 24.8% of all adults 50 years and older. A slightly higher proportion of the 50- to 64-year olds lived together with a partner or spouse (17.7%). Adults at CFCS level I more often lived with a partner (14.6%), than those at CFCS levels II–V. Having a more severe disability was associated with a decreased probability of having a partner. Only 4.9% of the individuals at GMFCS levels IV and V, and 1.3% at CFCS levels IV and V had a partner (Table 2).

**Table 2:** Adults who are single or have a partner, relative to their sex, age, GMFCS and CFCS levels.

		**Single**	**Partner who**	**Domestic partner**	
			**lives elsewhere**		***p*-value**
		***n* = 1551**	***n* = 101**	***n* = 140**	
		***N* (%)**	***N* (%)**	***N* (%)**	
**Sex**					
	Male	874 (90.0)	44 (4.5)	53 (5.5)	<0.001
	Female	677 (82.5)	57 (6.9)	87 (10.6)	
	Total	1,551 (86.6)	101 (5.6)	140 (7.8)	
**Age**					
	≤ 19 y	318 (97.8)	6 (1.8)	1 (0.3)	<0.001
	20–24 y	468 (89.8)	28 (5.4)	25 (4.8)	
	25–29 y	278 (84.5)	15 (4.6)	36 (10.9)	
	30–39 y	259 (81.4)	29 (9.1)	30 (9.4)	
	40–49 y	125 (77.2)	13 (8.0)	24 (14.8)	
	50–64 y	93 (75)	9 (7.3)	22 (17.7)	
	≥ 65 y	10 (76.9)	1 (7.7)	2 (15.4)	
	Total	1,551 (86.6)	101 (5.6)	140 (7.8)	
**GMFCS**					
	I	308 (76.8)	36 (9.0)	57 (14.2)	<0.001
	II	316 (82.1)	26 (6.8)	43 (11.2)	
	III	225 (84.0)	19 (7.1)	24 (9.0)	
	IV	296 (91.4)	15 (4.6)	13 (4.0)	
	V	406 (98.1)	5 (1.2)	3 (0.7)	
	Total	1,551 (86.6)	101 (5.6)	140 (7.8)	
**CFCS**					
	I	588 (76.1)	72 (9.3)	113 (14.6)	<0.001
	II	200 (91.7)	11 (5.0)	7 (3.2)	
	III	201 (91.8)	8 (3.7)	10 (4.6)	
	IV	212 (98.6)	1 (0.5)	2 (0.9)	
	V	252 (98.8)	2 (0.8)	1 (0.4)	
	Total	1,453 (86.5)	94 (5.6)	133 (7.9)	

*CFCS, Communication Function Classification System; GMFCS, Gross Motor Function Classification System; y, years*.

Age increased the likelihood of having a partner (OR 1.03, 95% CI 1.01–1.04), and women were almost twice as likely to have a partner as men (OR 1.89, 95% CI 1.36–2.63), when adjusted for GMFCS, CFCS, housing and occupation (Table 3). Adults at GMFCS level III were almost half as likely to have a partner (OR 0.45, 95% CI 0.27–0.74) compared with adults at GMFCS level I, and those at GMFCS level V were much less likely to have a partner (OR 0.12, 95% CI 0.04–0.31). Having more severe challenges with communication decreased the likelihood of having a partner, CFCS levels IV (OR 0.15, 95% CI 0.05–0.49), and V (OR 0.18; 95% CI 0.05–0.65) compared with adults at CFCS level I. Independent living (OR 5.01, 95% CI 3.30–7.59), and having a competitive employment (OR 1.67, 95% CI 1.15–2.43) increased the likelihood of having a partner (Table 3).

**Table 3:** Binary logistic regression analyses for having a partner presented as odds ratios (ORs) with 95% confidence intervals (CIs).

		**Having a partner**			
		**OR**	**95% CI**		***p*-value**
			**Lower**	**Upper**	
	**Age**	1.03	1.01	1.04	<0.001
**Sex**	Male	*ref*			
	Female	1.89	1.36	2.63	<0.001
**GMFCS**	I	*ref*			
	II	0.61	0.40	0.94	0.026
	III	0.45	0.27	0.74	0.002
	IV	0.31	0.18	0.55	<0.001
	V	0.12	0.04	0.31	<0.001
**CFCS**	I	*ref*			
	II	0.48	0.27	0.85	0.012
	III	0.66	0.37	1.21	0.179
	IV	0.15	0.05	0.49	0.002
	V	0.18	0.05	0.65	0.009
**Housing**	No independent living	*ref*			
	Independent living	5.01	3.30	7.59	<0.001
**Occupation**	No competitive employment	*ref*			
	Competitive employment	1.67	1.15	2.43	0.008

*All values are adjusted for all other variables in the model*.

*CFCS, Communication Function Classification System; GMFCS, Gross Motor Function Classification System*.

### Type of Housing

Most adults either lived with their parents (43.3%) or in independent living (41.3%), whereas 13.1% had assisted-living facilities. Females more often lived independently than males (44.9 vs. 38.4%), while males more often lived with their parents (45.5 vs. 40.7%) (Table 4). Housing differed significantly between age groups (*p* <0.001). Most of the 25- to 29-year olds (55.6%) lived independently, increasing up to 72.4% of the 40- to 49-year olds. Most of the younger adults lived at home with their parents (91.3% of those under 20 years and 63.4% of the 20- to 24-year olds). There was also a relatively large number of individuals, 27.8% aged 25–29 years, who still lived at home. The number of individuals living in assisted-living facilities increased with age, ranging from 2% for those under 19 years, up to 31.2% for those over 50 years of age (Table 4).

**Table 4:** Type of housing and personal assistance (hours/week) for all adults relative to their sex, age, GMFCS and CFCS levels.

			**Housing**					**Personal assistance, h/w**			
		**Independent**	**With parents**	**Assisted living**	**Other**	***p*-value**	**No**	** <60**	**60–160**	**>160**	***p*-value**
		***N* (%)**	***N* (%)**	***N* (%)**	***N* (%)**		***N* (%)**	***N* (%)**	***N* (%)**	***N* (%)**	
**Sex**						0.018					0.993
	Male	386 (38.4)	457 (45.5)	134 (13.3)	28 (2.8)		539 (55.3)	59 (6.1)	151(15.5)	225 (23.1)	
	Female	372 (44.9)	337 (40.7)	107 (12.9)	13 (1.6)		448 (54.6)	51 (6.2)	129 (15.7)	192 (23.4)	
	Total	758 (41.3)	794 (43.3)	241 (13.1)	41 (2.2)		987 (55.0)	110 (6.1)	280 (15.6)	417 (23.2)	
**Age**						<0.001					<0.001
	≤ 19 y	7 (2.0)	315 (91.3)	7 (2.0)	16 (4.6)		181 (56.4)	41 (12.8)	61 (19.0)	38 (11.8)	
	20–24 y	136 (25.5)	338 (63.4)	44 (8.3)	15 (2.8)		302 (57.3)	33 (6.3)	95 (18.0)	97 (18.4)	
	25–29 y	184 (55.6)	92 (27.8)	48 (14.5)	7 (2.1)		151 (45.8)	14 (4.2)	41 (12.4)	124 (37.6)	
	30–39 y	224 (69.1)	41 (12.7)	58 (17.9)	1 (0.3)		162 (50.3)	11 (3.4)	46 (14.3)	103 (32.0)	
	40–49 y	118 (72.4)	6 (3.7)	38 (23.3)	1 (0.6)		89 (56.3)	4 (2.5)	24 (15.2)	41 (25.9)	
	50–64 y	83 (66.4)	2 (1.6)	39 (31.2)	1 (0.6)		91 (73.4)	7 (5.6)	13 (10.5)	13 (10.5)	
	≥ 65 y	6 (46.2)	0	7 (53.8)	0		11 (91.7)	0	0	1 (8.3)	
	Total	758 (41.3)	794 (43.3)	241 (13.1)	41 (2.2)		987 (55.0)	110 (6.1)	280 (15.6)	417 (23.2)	
**GMFCS**						<0.001					<0.001
	I	167 (40.2)	213 (51.3)	27 (6.5)	8 (1.9)		414 (97.6)	6 (1.4)	1 (0.2)	3 (0.7)	
	II	158 (40.6)	151 (38.8)	68 (17.5)	12 (3.1)		302 (79.5)	27 (7.1)	28 (7.4)	23 (6.1)	
	III	127 (47.0)	92 (34.1)	41 (15.2)	10 (3.7)		138 (53.5)	35 (13.6)	57 (22.1)	28 (10.9)	
	IV	142 (42.4)	147 (43.9)	39 (11.6)	7 (2.1)		69 (21.2)	31 (9.5)	105 (32.3)	120 (36.9)	
	V	164 (38.6)	191 (44.9)	66 (15.5)	4 (0.9)		64 (15.7)	11 (2.7)	89 (21.9)	243 (59.7)	
	Total	758 (41.3)	794 (43.3)	241 (13.1)	41 (2.2)		987 (55.0)	110 (6.1)	280 (15.6)	417 (23.2)	
**CFCS**						<0.001					<0.001
	I	413 (51.3)	314 (39.0)	54 (6.7)	24 (3.0)		642 (80.9)	43 (5.4)	60 (7.6)	49 (6.2)	
	II	74 (33.2)	101 (45.1)	41 (18.4)	7 (3.1)		128 (59.0)	15 (6.9)	41 (18.9)	33 (15.2)	
	III	75 (33.6)	109 (48.9)	37 (16.6)	2 (0.9)		61 (29.2)	20 (9.6)	54 (25.8)	74 (35.4)	
	IV	66 (30.3)	102 (46.8)	48 (22.0)	2 (0.9)		57 (27.1)	15 (7.1)	61 (29.0)	77 (36.7)	
	V	93 (34.7)	119 (44.4)	53 (19.8)	3 (1.1)		52 (20.3)	9 (3.5)	43 (16.8)	152 (59.4)	
	Total	721 (41.5)	745 (42.9)	233 (13.4)	38 (2.2)		940 (55.8)	102 (6.0)	259 (15.4)	385 (22.8)	

*CFCS, Communication Function Classification System; GMFCS, Gross Motor Function Classification System; y, years*.

Type of housing differed between adults at different GMFCS and CFCS levels (*p* <0.001). Most adults at CFCS level I lived independently (51.3%), while the majority of those at CFCS levels II to V lived with their parents (44.4–48.9%). Assisted-living facilities were most frequent in adults at GMFCS levels II (17.5%), and V (15.5%). Most individuals at GMFCS level I either lived with their parents (51.3%) or in independent living (40.2%). The proportion of adults living independently was almost equal at GMFCS levels I (40.2%) and V (38.6%), despite the differences in motor functioning and ability to manage daily life (Table 4).

The single most important factor for independent living was access to personal assistance (Table 5). Personal assistance for >160 h/w increased the likelihood of independent living over 50 times (OR 57.07, 95% CI 32.35–101). In addition, increasing age, having a partner, and being employed increased the probability of independent living (Table 5).

**Table 5:** Binary logistic regression analyses for independent living and competitive employment presented as odds ratios (ORs) with 95% confidence intervals (CIs).

		**Independent living**				**Competitive employment**			
		**OR**	**95% CI**		***p*-value**	**OR**	**95% CI**		***p*-value**
			**Lower**	**Upper**			**Lower**	**Upper**	
	**Age**	1.12	1.10	1.13	<0.001	1.02	1.00	1.03	0.074
**Sex**	Male	*ref*				*ref*			
	Female	1.21	0.93	1.59	0.160	0.54	0.39	0.76	<0.001
**GMFCS**	I	*ref*				*ref*			
	II	1.05	0.68	1.62	0.834	0.73	0.48	1.12	0.147
	III	0.51	0.30	0.86	0.011	0.54	0.33	0.87	0.013
	IV	0.32	0.18	0.58	<0.001	0.43	0.25	0.75	0.003
	V	0.36	0.19	0.68	0.002	0.17	0.06	0.51	0.002
**CFCS**	I	*ref*				*ref*			
	II	0.31	0.19	0.50	<0.001	0.36	0.20	0.65	0.001
	III	0.15	0.09	0.25	<0.001	0.13	0.05	0.34	<0.001
	IV	0.13	0.07	0.22	<0.001	0.07	0.02	0.32	<0.001
	V	0.14	0.08	0.24	<0.001	0.00	–	–	0.994
**Partner**	Single	*ref*				*ref*			
	Partner	6.21	3.96	9.74	<0.001	1.65	1.12	2.42	0.011
**Housing**	No independent living	–	–	–	–	*ref*			
	Independent living	–	–	–	-	5.51	3.78	8.63	<0.001
**Occupation**	No competitive employment	*ref*				–	–	–	–
	Competitive employment	5.21	3.42	7.93	<0.001	–	–	–	–
**Personal assistance**	No assistance	*ref*							
	<60 h/w	1.11	0.53	2.34	0.780	–	–	–	–
	60–160 h/w	10.03	5.89	17.08	<0.001	–	–	–	–
	>160 h/w	57.07	32.25	101.0	<0.001	–	–	–	–

*All values are adjusted for all other variables in the models*.

*CFCS, Communication Function Classification System; GMFCS, Gross Motor Function Classification System*.

### Personal Assistance

In total, 807 adults (45%) had personal assistance, at similar levels for males and females (Table 4). Most individuals had assistance >160 h/w (23.2%), ranging from 10.5% of those over 50 years, up to 37.6% of the 25- to 29-year olds. The proportion of adults who received personal assistance increased with increasing GMFCS and CFCS levels (*p* <0.001). Most adults at GMFCS levels I (97.6%) and II (79.5%) had no assistance. The amount of assistance increased proportionally for both GMFCS levels IV and V, such that 59.7% at level V had assistance over 160 h/w. More individuals at CFCS levels I received assistance at all assistance levels, compared with GMFCS levels I. Those with fewer communication skills also had personal assistance for more hours per week than those with more ability, with 59.4% of the adults at CFCS level V having assistance more than 160 h/w, compared with 35.4% at CFCS level III and 6.2% of those at CFCS level I (Table 4).

### Occupation

The majority had their primary occupation at an activity centre for people with intellectual disabilities (36.7%). Because of the high number of young adults in the population, many individuals either went to mainstream education/higher education or attended special school (19.4 vs. 10.1%) (Table 6). In the age span 20–64 years, 17.5% had competitive employment, 3.7% had supported employment and 45.2% attended activity centres. In the younger age group up to 24 years old, 36.9% went to mainstream education and 20.5% to special schools. Individuals at activity centres were found in all age groups. Competitive employment ranged from 0.6% of those under 19 years, up to 26.1% of the 40- to 49-year olds, then declined (Table 6).

**Table 6:** Primary occupation and full-time or part-time occupation for all adults with CP relative to their sex, age, GMFCS, and CFCS levels.

		**Education**		**Employment**		**Activity**	**No**		**Full or part-time**		
		**Mainstream**	**Special**	**Competitive**	**Supported**	**Centre**	**Occupation**	***p*-value**	**Full time**	**Part-time**	***p*-value**
		***n* = 355**	***n* = 184**	***n* = 261**	***n* = 55**	***n* = 672**	***n* = 299**		***n* = 795**	***n* = 656**	
		***N* (%)**	***N* (%)**	***N* (%)**	***N* (%)**	***N* (%)**	***N* (%)**		***N* (%)**	***N* (%)**	
**Sex**								0.372			0.323
	Male	181 (18.1)	103 (10.3)	156 (15.6)	29 (2.9)	371 (37.2)	158 (15.8)		446 (56.0)	351 (44.0)	
	Female	174 (21.0)	81 (9.7)	105 (12.7)	26 (3.1)	301 (36.3)	141 (17.0)		349 (53.4)	305 (46.6)	
	Total	355 (19.4)	184 (10.1)	261 (14.3)	55 (3.0)	672 (36.8)	299 (16.4)		795 (54.8)	656 (45.2)	
**Age**								<0.001			<0.001
	≤ 19 y	181 (52.6)	140 (40.7)	2 (0.6)	0 (0.0)	6 (1.7)	15 (4.4)		283 (91.3)	27 (8.7)	
	20–24 y	142 (26.7)	39 (7.3)	65 (12.2)	20 (3.8)	187 (35.2)	78 (14.7)		253 (59.1)	175 (40.9)	
	25–29 y	20 (6.0)	2 (0.6)	64 (19.3)	11 (3.3)	182 (54.8)	53 (16.0)		98 (36.6)	170 (63.4)	
	30–39 y	9 (2.8)	2 (0.6)	61 (18.9)	13 (4.0)	175 (54.3)	62 (19.3)		95 (38.2)	154 (61.8)	
	40–49 y	2 (1.2)	0	42 (26.1)	9 (5.6)	70 (43.5)	38 (23.6)		42 (35.9)	75 (64.1)	
	50–64 y	1 (0.8)	1 (0.8)	27 (22.0)	2 (1.6)	50 (40.7)	42 (34.1)		24 (31.2)	53 (68.8)	
	≥ 65 y	0	0	0	0	2 (15.4)	11 (84.6)		0	2 (100)	
	Total	355 (19.4)	184 (10.1)	261 (14.3)	55 (3.0)	672 (36.8)	299 (16.4)		795 (54.8)	656 (45.2)	
**GMFCS**								<0.001			<0.001
	I	162 (39.2)	20 (4.8)	113 (27.4)	16 (3.9)	57 (13.8)	45 (10.9)		251 (70.5)	105 (29.5)	
	II	70 (18.0)	33 (8.5)	70 (18.0)	11 (2.8)	152 (39.1)	53 (13.6)		164 (51.6)	154 (48.4)	
	III	40 (14.9)	26 (9.7)	45 (16.8)	9 (3.4)	91 (34.0)	57 (21.3)		104 (52.3)	95 (47.7)	
	IV	48 (14.5)	39 (11.8)	29 (8.8)	12 (3.6)	134 (40.6)	68 (20.6)		126 (50.4)	124 (49.6)	
	V	35 (8.2)	66 (15.5)	4 (0.9)	7 (1.6)	238 (55.9)	76 (17.8)		150 (45.7)	178 (54.3)	
	Total	355 (19.4)	184 (10.1)	261 (14.3)	55 (3.0)	672 (36.8)	299 (16.4)		795 (54.8)	656 (45.2)	
**CFCS**								<0.001			0.015
	I	234 (29.1)	26 (3.2)	228 (28.4)	37 (4.6)	126 (15.7)	153 (19.0)		359 (57.3)	268 (42.7)	
	II	54 (24.7)	21 (9.6)	17 (7.8)	7 (3.2)	89 (40.6)	31 (14.2)		105 (57.7)	77 (42.3)	
	III	25 (11.2)	27 (12.1)	5 (2.2)	5 (2.2)	132 (58.9)	30 (13.4)		90 (50.3)	89 (49.7)	
	IV	13 (6.0)	42 (19.4)	2 (0.9)	1 (0.5)	132 (61.1)	26 (12.0)		95 (53.4)	83 (46.6)	
	V	13 (4.9)	44 (16.5)	0	2 (0.7)	158 (59.2)	50 (18.7)		98 (48.0)	106 (52.0)	
	Total	339 (19.6)	160 (9.2)	252 (14.6)	52 (3.0)	637 (36.8)	290 (16.8)		747 (54.5)	623 (45.5)	

*CFCS, Communication Function Classification System; GMFCS, Gross Motor Function Classification System; y, years*.

Occupation also differed significantly between adults at different GMFCS and CFCS levels (Table 6). The majority of individuals in mainstream education were classified at GMFCS levels I (39.2%) or II (18.0%), with the same tendency for CFCS levels, with individuals at levels I (29.1%) and II (24.7%). The opposite was seen for those attending a special school, mostly including individuals at GMFCS levels IV (11.8%) and V (15.5%) and also at CFCS levels IV (19.4%) and V (16.5%). Competitive employment was more common in individuals at GMFCS level I (27.4%) and CFCS level I (28.4%), thereafter decreasing proportionally for increasing GMFCS/CFCS levels. Having no occupation was evenly distributed among the different GMFCS and CFCS levels (Table 6).

Independent living (OR 5.51, 95% CI 3.78–8.63) and having a partner (OR 1.65, 95% CI 1.12–2.42) increased the likelihood of having competitive employment, whereas women were half as likely to have employment as men (OR 0.54, 95% CI 0.39–0.76). Reduced communication ability seemed to be the highest risk factor for not having employment, ranging from CFCS level II (OR 0.36, 95% CI 0.2–0.65) to level IV (OR 0.07, 95% CI 0.02–0.32). No individual at CFCS V had a competitive employment (Table 5).

### Full or Part-Time Occupation

Most individuals (54.8%) worked full time (>30 h/w). There were no significant differences between males and females in worked h/w. A full-time occupation was most common for the youngest age group of 16 to 19 years attending school (91.3%). Considering individuals of working age (20–64-year olds), full-time occupation gradually decreased from 59.1% in the 20- to 24-year olds to 31.2% of the 50- to 64-year olds, whereas part-time work increased successively for 20- to 24-year olds, to peak at 50 to 64 years of age. Two individuals over 65 years worked part-time (Table 6).

Full-time and part-time occupation differed significantly between GMFCS (*p* <0.001) and CFCS levels (*p* = 0.015). Most individuals at GMFCS level I, (70.5%) had a full-time occupation. More individuals at CFCS level I (42.7%) worked part-time than those at GMFCS level I (29.5%). The largest groups having a part-time occupation were adults at GMFCS level V and CFCS level V (54.3 vs. 52%) (Table 6).

## Discussion

This study describes the living conditions and social outcomes in 1,888 adults with CP, aged 16–78 years old. Only one in eight had a partner, and one in six of the 20- to 64-year olds had competitive employment, revealing that reduced communication ability seemed to be the highest risk factor for not having employment. Access to personal assistance was the single most important factor for independent living and explained why independent living was almost as common in adults with the least and the most severe motor impairments.

### Having a Partner

To have support, but also help from family and friends, is considered the main core for gaining quality of life (20). We found that only one in eight people in our study had a partner (12.7%). This is slightly lower than a previous report that found 22% had a partner (10). The higher prevalence might be explained by different inclusion criteria because the authors excluded individuals with learning disabilities and those living in assisted-living facilities (10). In a recent Danish study (12), as much as 28% of the 416 adults with CP had a partner. The population in that study had a slightly higher mean age of 32 years, than the mean age of 25 years in our cohort. We found that older age increased the likelihood of having a partner. Another study of 61 young adults with CP (20–22 years), reported that 5% lived with a partner (6). This is in line with our findings, where 4.8% (20–24 y), lived with a partner or spouse.

A novel but unexpected finding was that women were almost twice as likely to have a partner as men, either a domestic partner or a partner living elsewhere. Adults at CFCS level I and more often lived with a partner than those with less communication ability. This is in line with previous findings where better CFCS has been associated with having experience of intimate relationships, although, unlike our study, the authors found no association with the GMFCS (6). We found that the severity of CP, both in terms of communication and gross motor function decreased the likelihood of having a partner. The difference in findings can perhaps be explained by their smaller sample size, slightly different outcomes, and analyses of data. The link between the quality of life and social relations (family, friends, and others), affects an individual's life, both positively and negatively depending on the characteristic of the relationship (20). Our findings also reinforce the crucial need for good communication abilities, particularly important for social functioning in young adults with CP (6, 12). Unfortunately, having a partner seems to be less common in adults with CP than in the general population.

### Type of Housing

To have independent living is a prioritised goal for young adults (5). We found that most adults either lived with their parents (43.3%) or in independent living (41.3%), whereas only 13.1% lived in assisted-living facilities. There was a clear association between age and type of housing. We found that many younger adults in the transition years still lived with their parents, which agrees with other studies (21, 22), whereas as many as 72.4% in the age group 40–49 years lived independently. Other studies report that between 13 and 95% live with their parents, and between 28 and 86% of adults with CP live independently (10, 12, 21–24). It is important to note that the selection criteria, sample sizes, age and degree of severity often differ substantially among these studies, making it difficult to compare the results.

Almost half of the young adults with CP rely on family members for help with ADL on a daily basis (6) and this may be one of the reasons why we found that almost one-third of individuals aged 25–29 years still lived at home. Our data represent a nationwide cohort, and this may be another reason for the differences related to housing. The 61 young adults in the study by Jacobson et al. (6) live in a large city, with a tough housing market. This may explain why only 20% of young adults had moved away from their parents, compared with 44% of age-matched adults from official statistics (6). Today, as a young adult (with or without a disability), financial constraints can make entering the housing market a challenge, which may explain the higher number of young individuals still living at home. Access to, and quality of, assisted-living facilities can also play a part in why young adults remain so long in the parental home, especially when the parents want to find a facility that matches their expectations. Instead, young adults remain with their parents (12). To care for a person with impairments can be stressful, particularly for the parents and especially as challenging behaviour often occurs in the context of cognitive impairment and mental health problems, in addition to the individuals' physical impairments (25). In addition, the parents' health and well-being are of great importance when planning for youth transition into adulthood (4).

We found that males more often live with their parents or in assisted-living facilities than females who more often lived independently, while others did not find any differences between males and females regarding the type of housing (12). This difference might be explained by our larger cohort (1,888 vs. 416). In line with previous studies (11, 12, 21, 26), we found that one in five adults lived in assisted-living facilities, most frequently for adults at GMFCS levels II and V. This observation is most likely explained by the higher proportion of individuals attending special schools or activity centres for these two groups, indicating cognitive disabilities.

Like the findings by Michelsen et al. (12), we found that the type of housing also differed between adults at different levels of motor function and communication ability. An unexpected finding was that independent living levels were almost equal in adults at GMFCS levels I and V. When adjusting for several factors in the regression analysis, adults at GMFCS V were much less likely to live independently than adults at GMFCS I. However, this seemed to be compensated by their access to personal assistance. We identified personal assistance as the single most important factor for independent living. Access to personal assistance for >160 h/w, increased the likelihood of independent living more than 50-fold. In addition, increasing age, having a partner and employment increased the probability of independent living. Obtaining personal assistance opens possibilities and increases social integration and participation for adults with CP, giving reason to live an independent life in the community.

### Personal Assistance

Some form of personal assistance is currently available in all Nordic countries, most Western European countries, Australia, parts of Asia, the U.S. and Canada (25). In 2017 there were 19,690 persons receiving personal assistance in Sweden. For the last three decades, quality of life has increased, for those gaining assistance. Today, the future is uncertain, as the state in many cases reduces, or denies assistance hours (27).

We found that 45% of our adult population with CP received personal assistance, and one out of five had assistance more than 160 h per week. In a study from 2014, 49% of 102 young adults had personal assistance (11) and in a study from 2001, 55% received assistance. These studies included individuals with CP, but without cognitive disabilities (10). The actual difference in access to personal assistance over these 20 years may be even more pronounced, considering that those with cognitive disabilities were included in our study but excluded in the study from 2001.

The access to personal assistance was similar for males and females (44.7 vs. 45.4%). This differs from Sweden as a whole, where a slight predominance of males (54%) had personal assistance (27). The reverse is seen in the U.S. where there is a predominance of assistance for females (65%) (25). In addition, younger adults received more hours of assistance than older adults, with a peak for those aged 25–29 years. Age may be an explaining factor for the reduced assistance frequency (11), because the current population is older than in 2014. Another scenario could be that government-granted funds for assistance have decreased (27) for the population of adults with CP.

More individuals at CFCS levels I received assistance than those at GMFCS levels I. A plausible explanation is that even those with severe motor disabilities can have better communication skills than gross motor skills. Assistance increased with higher GMFCS levels, and to gain access to assistance, the individual had to fulfil certain criteria. In addition to the medical diagnoses that are required, they also needed assistance for more than 20 h/w for activities of daily living, on at least one of the following basic needs: personal hygiene, eating, dressing, and undressing, communicating with others and other help. Limited participation in activities may negatively impact the quality of life, health and family functioning (25), which reinforces the need to find means to continue providing assistance to people with disabilities such as CP.

### Occupation, and Full or Part-Time Occupation

It has been stated that we need to identify factors for accessibility regarding occupation to minimise the negative effect of impairment, such as CP (12). Almost one in five adults in the age range of 20–64 years had competitive employment, which is the same as in young adults in 2014 (11). This figure is much lower than that reported for 416 Danish adults with CP, (29–35 years), where 33% had competitive employment (12). According to Mesterman et al. (21), 23% had competitive employment while two out of three received monthly disability support. A large systematic review and meta-analysis by van Gorp et al. (23) concluded that on average, 40% had remunerative employment. The difference in employment rates could be due to the difference in populations studied, sample sizes, social constructs, and regulations. Even so, the low number of Swedish adults with CP and employment is discouraging.

Reduced communication ability was the highest risk factor for not having competitive employment. Living with a partner almost doubles the likelihood of having competitive employment, whereas women were half as likely to have employment as men. Most individuals worked full time. We found no significant differences between men and women regarding full- or part-time occupation, while Dutch women with CP showed a strong decline in working hours, especially when becoming a mother (22). Looking at all types of work and sexes for those in working age (20–64-year olds), full-time occupation gradually decreased, whereas part-time work successively increased, with older age (12). Having a more severe disability increased the likelihood of individuals with CP working part-time, with GMFCS/CFCS level V as the largest group. More individuals at CFCS level I worked part-time than those at GMFCS level I. As mentioned earlier, several adults at higher GMFCS levels (lower motor function), had good communication abilities (CFCS level I).

The majority aged 20–64 years had their primary occupation at activity centres (45.2%). This is similar to the 41% reported by Jacobson et al. (6). but contrasts with the findings from a Dutch longitudinal study (22), where only one in five had their occupation at activity centres. Even though Benner et al. (22) describe the Dutch labour market as having, “high density of sheltered employment and financial resources”, numbers are still lower than in our study. A postal survey in Israel by Mesterman et al. (21), revealed that only 15% had their occupation at activity centres. As both the current study and that of Jacobson et al. (6) originate from Sweden, the explanation may lie in the different social systems. Perhaps the Swedish welfare system offers sheltered work (activity centres), to a much higher extent than in other countries.

Because of the high number of young adults in the population, many individuals either went to mainstream education or attended a special school. Full-time occupation was most common for the youngest age group of 16–19 years that attended school. Lack of social competence (28), physical fatigue (29) and increased ageing (30) are different factors mentioned when discussing the lack of individuals with CP engaged in competitive employment, or employment overall. Addressing psychosocial issues and educational and vocational needs is necessary when considering social outcomes. It is important to open the debate with medical experts, legislators, and politicians to make some changes.

### Limitations

There are several limitations to this study. The cross-sectional design was used to document the status of a group at a particular point in time, which means that we can show association but not any causal relationships. Another limitation is the skewed distribution of ages because four out of five individuals were younger than 40 years of age, also mentioned earlier in an initial study of a smaller cohort in 2014 (11). The GMFCS levels are also skewed because the study mostly included individuals at GMFCS levels I and V. In children, GMFCS level I usually represents up to 40% of the population (9). This could be explained by the premature decline in gross motor function seen in adults with CP (31), or a selection bias where more adults with severe motor impairments agree to participate in the continuous follow-up programme. Another limitation is that we had no access to data on individual social competence, potentially affecting cohabitation (28) or factors influencing personal assistance, such as behavioural problems, autism disorders and visual impairment.

Nevertheless, the strength of this study is the large study population, which allows for differentiation and comparisons of all levels of gross motor function and communication ability, and between sexes and age groups. Regular systematic and uniform assessments assure high accountability.

## Conclusions

Only one in eight adults with CP have a partner, and one in six have competitive employment. Access to personal assistance is the single most important factor for independent living.

The opportunity for equality is an important issue regarding living conditions and social outcomes such as independent living, having employment and finding a partner. However, for adults with CP, the possibility to be active and to participate, and the contextual factors in the individual's life, may affect all aspects of their life. Our primary goal should be to support adults with CP throughout their lifetime to allow them to achieve the best possible outcomes in all aspects of life.

## Data Availability Statement

The data analysed in this study was obtained from the Cerebral Palsy Follow-Up Program (CPUP) registry, the following licences/restrictions apply: requests to access the datasets are subject to ethical approval and must first be granted by KVB Region Skåne. Requests to access these datasets should be directed to https://vardgivare.skane.se/kompetens-utveckling/forskning-inom-region-skane/utlamnande-av-patientdata-samradkvb/.

## Ethics Statement

The studies involving human participants were reviewed and approved by Regional Ethical Review Board, Lund, Sweden. Written informed consent from the participants' legal guardian/next of kin was not required to participate in this study in accordance with the national legislation and the institutional requirements.

## Author Contributions

ER-B collected the data and supported the analysis and interpretation of data and actively revised the manuscript. KP performed the analysis and drafted the manuscript. Both authors designed the study and contributed to the article and approved the submitted version.

## Funding

This study was supported by grants from FORTE—the Swedish Research Council for Health, Working Life and Welfare, grant no: 2018-01468.

## Conflict of Interest

The authors declare that the research was conducted in the absence of any commercial or financial relationships that could be construed as a potential conflict of interest.

## Publisher's Note

All claims expressed in this article are solely those of the authors and do not necessarily represent those of their affiliated organizations, or those of the publisher, the editors and the reviewers. Any product that may be evaluated in this article, or claim that may be made by its manufacturer, is not guaranteed or endorsed by the publisher.
